# A case of quadriplegia with gastric perforation

**DOI:** 10.4103/0974-2700.66562

**Published:** 2010

**Authors:** Sankalp Dwivedi, Amit Agrawal, Manisha Bhatt, Surya Pratap Singh

**Affiliations:** Department of Surgery, Datta Meghe Institute of Medical Sciences, Sawangi (Meghe), Wardha, India; 1 Department of Neurosurgery, Datta Meghe Institute of Medical Sciences, Sawangi (Meghe), Wardha, India; 2 Department of Anesthesiology, Datta Meghe Institute of Medical Sciences, Sawangi (Meghe), Wardha, India

**Figure 1:**
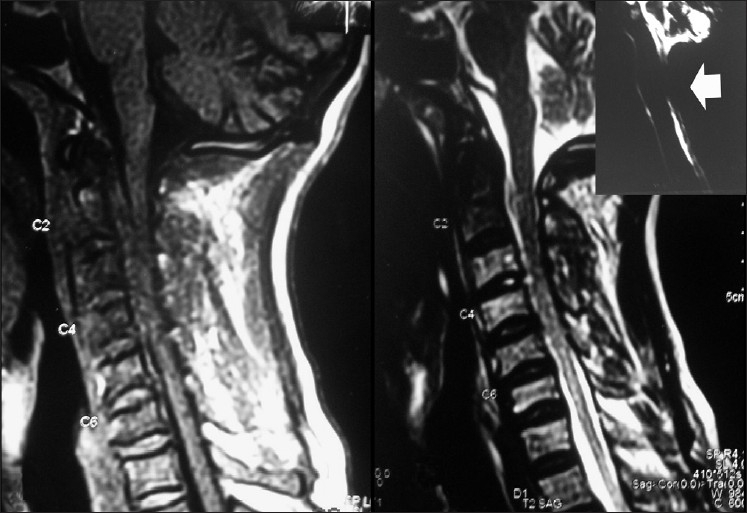
Magnetic resonance imaging of the cervical spine T1W and T2W sagittal images showing C3-5 cord compression

Patients with cervical cord lesions have an increased susceptibility of developing life-threatening gastrointestinal complications.[1–5] The reported incidence of gastrointestinal tract complications in spinal cord injury patients ranges from 4.7%[3] to 6.2%.[1] A 45-year-old gentleman was admitted in the critical care unit with the complaints of progressive quadriparesis of 2 weeks duration. He was bedridden and was on an indwelling urinary catheter for the last 7 days. His general and systemic examination was unremarkable. Neurologically, higher mental functions and cranial nerve examination were normal. He had hypotonia in all four limbs, sensory loss to all modalities below C5 and grade 1-2/5 power in the upper and lower limbs. Deep tendon reflexes were sluggish in both upper and lower limbs. Bilateral planters were extensor. X-ray of the cervical spine was normal. Magnetic resonance imaging of the cervical spine showed diffuse cord compression (C3-5 level) with signal intensity changes [Figure 1].

**Figure 2:**
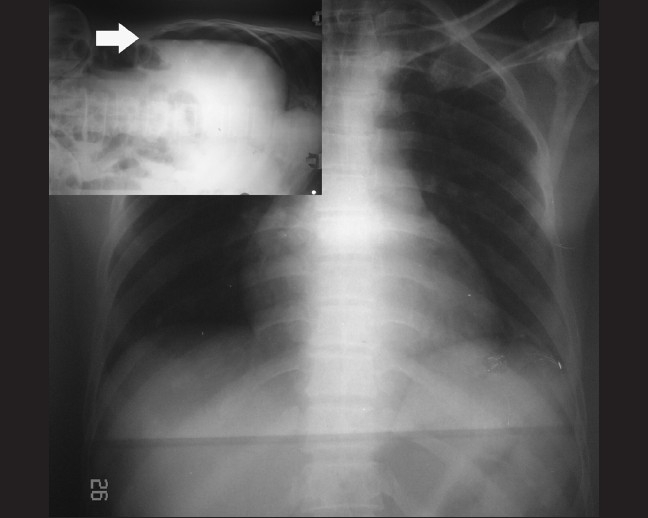
X-ray of the chest and upper abdomen with both the domes of the diaphragm appearing apparently normal. However, X-ray of the left lateral decubitus showed free gas in the peritoneum (inset, arrow)

Blood investigations, including hemoglobin, total leukocyte and differential counts, were within the normal limit, except a raised erythrocyte sedimentation rate. Mantoux test was positive. The patient was managed conservatively and was on a low dose of steroids. On the third post-admission day, the patient developed hypotension (blood pressure not recordable, pulse not palpable) and had increased motor weakness. The patient became drowsy. Chest and detailed per-abdomen examinations were normal. Clinically, a possibility of worsening in cervical cord edema with resultant spinal shock was suspected. Accordingly, under the cover of proton pump inhibitors, the dose of steroid was escalated and the patient was resuscitated with intravenous fluids and kept nil by mouth. The patient gradually became alert and the pulse and blood pressure became normal. However, after 48 h, he started developing abdominal distension and respiratory distress. Per-abdominal examination revealed no guarding, rigidity or rebound tenderness. Liver dullness was obliterated and bowel sounds were absent. Based on these findings, a diagnosis of perforation peritonitis was suspected and a nasogastric tube was inserted. As the patient was quadriplegic and bedridden, a supine X-ray chest and abdomen could be performed and it was non-contributory; however, an X-ray abdomen in the lateral decubitus (after pushing 100 cc air through the nasogastric tube) showed free air in the peritoneal cavity and diagnosis of perforation of hollow viscous was made [Figure 2]. Previous history related to peptic ulcer disease was non-contributory. Repeat blood examination showed polymorphonuclear leucocytosis, with a total count of 12,000/mm^3^. The patient underwent emergency laparotomy and repair of a pre-pyloric 0.5 cm × 0.5 cm anterior wall peptic perforation.

The use large-dose steroid administration has been advocated in spine-injured patients to lessen neurologic deficits; however, it can act as a two-edged sword[3,4] as there is an increase in the incidence of hemorrhaging and perforating gastrointestinal lesions in patients with cervical cord lesions,[2,3,5] particularly in patients with complete deficits.[3] As in the present case, patients with complete high cervical cord lesions can develop painless perforation and peritonitis, with increased morbidity.[2] As in the present case, in the background of acute spinal cord lesion, clinical manifestations of silent life-threatening acute abdominal complication may be masked by the associated motor and sensory deficits. In the present case, it was not possible to diagnose whether the gastric perforation was because of the use of steroids or was an unusual complication of Cushing’s ulcer in a patient of spinal cord lesion. As in the literature, we recommend that a high index of suspicion and an aggressive therapeutic approach is necessary to avoid an increase in morbidity.[2,3] In summary, when there is a hollow viscous perforation, it is straightforward and quite easy to diagnose based on clinical and radiological findings. However, when routine X-ray of the abdomen is inconclusive, a lateral X-ray of the abdomen after insufflation of the 100 cc air through the nasogastric tube can help in the diagnosis without the further need of computed tomography scan of the abdomen.
